# Editorial: Neuropharmacology of neuro -degenerative, -logical, -psychiatric disorders

**DOI:** 10.3389/fphar.2023.1223200

**Published:** 2023-06-13

**Authors:** Harpreet Kaur

**Affiliations:** Center for Neurological Restoration, Neurological Institutes, Cleveland Clinic, Cleveland, OH, United States

**Keywords:** brain disorders, animal model, drug repositioning, drug toxicity, clinical research

Neuropsychological disorders are the leading cause of disability worldwide, significantly burdening health and the economy (GBD 2016 Neurology Collaborators, 2019). Despite the extensive clinical and experimental (*in-vivo* and *in-vitro*) research to reduce the burden, over the past 25 years, the incidence, prevalence, mortality, and disability rates of neurological disorders have been rising globally. In this Research Topic of Frontiers in Pharmacology on “*Neuropharmacology of Neuro -degenerative*, *-logical*, *-psychiatric disorders*” we received three original articles, one clinical trial, and one review covering recent and exciting findings about the role of the various promising drugs or potential compounds to treat these disorders.

Hecker et al. studied multiple sclerosis (MS), a chronic autoimmune disease, and screened three databases named Stockley’s, Drugs.com, and MediQ to find potential drug-drug interactions (pDDIs) among MS patients. Total 1,684 pDDIs were identified in 627 patients enrolled in this study, of which 318 pDDIs were common among the three databases. This article reported that 35.2% of the 627 patients had at least one severe pDDI, significantly associated with older age, lower educational level, living without a partner, comorbidities, and the number of medications taken. The author emphasizes addressing pDDIs through improved digital health services and recommends screening multiple databases to find pDDIs (Hecker et al.).

Another research article by Zhu et al. addresses structural abnormalities associated with depression using chronic unpredictable stress (CUS) animal model. This study’s findings suggest that the liver X receptor agonist, GW3965, improved CUS-induced depressive symptoms, improving understanding of underlying molecular mechanisms and behavioral basis. Further investigations revealed a decreased number of CC1+ oligodendrocytes (OL) and the density of BrdU+/Olig2+/CC1+ cells in each hippocampal subregion of CUS mice. GW3965 treatment in CUS mice increases MBP expression in the CA3 region. These findings indicate that improved OL maturation and enhancement of myelination may be structural mechanisms underlying the antidepressant effects of LXR agonists (Zhu et al.).

Li et al. studied the effect of duloxetine on anxiety and pain in complete Freund’s adjuvant mice with chronic inflammatory pain. Intraperitoneal injection of duloxetine causes decreased glutamatergic excitability and enhanced serotonin concentration in the anterior cingulate cortex. This study sheds light on the potential mechanism of the anxiolytic effect in chronic pain-induced anxiety and further opens avenues for novel drug development (Li et al.).

In a clinical trial among schizophrenia patients, Weng et al. explored the risk factors, clinical variables, and social functions associated with drug-induced parkinsonism (DIP) in schizophrenia patients of Chinese ethnicity. This study reported that age, treatment with a high dose of D2 receptor antagonistic, and valproate increases the risk of DIP. In this cohort, psychiatric symptoms and social dysfunction have been linked with DIP. Expectedly, old age was also associated with psychotic symptoms and interfered with the ability to function socially. The authors concluded that early intervention and treatment of DIP could improve the prognosis and social capabilities of schizophrenia patients (Weng et al.).

Latif et al. reviewed repositioned drugs those currently in clinical trials and approved for Parkinson’s Disease (PD). Parkinson’s disease is a progressive disorder that affects dopaminergic neurons in the substantia nigra. Drug repurposing is utilizing existing medications to treat diseases other than they were initially designed for. New agent development through repositioning seems promising as it is economical in terms of time and cost. Food Drug Administration (FDA) has approved five repositioned drugs for PD, emphasizing the importance of disease-modifying therapies for PD. This era of precision medicine integrated with delineating disease and drug action mechanisms further emphasizes the need and application of drug repositioning (Latif et al.).

Hopefully, these articles advance our understanding of potential molecular mechanisms and provide evidence for promising future drugs. Also, these articles help researchers to define their study design. Because of these compelling pieces of evidence, there is a pressing need to design more ambitious preclinical and clinical studies to determine the efficiency of drug therapy in various diseases.
